# The RANZCP Board rejects scrutiny of decisions that affect members

**DOI:** 10.1177/10398562231160359

**Published:** 2023-02-24

**Authors:** Andrew Amos

**Affiliations:** Townsville, QLD

Dear Sir,

The most damaging feature of the COVID-19 pandemic has been the rhetorical use of science to avoid scrutiny, contributing to a failure to identify and correct weaknesses which added massive unnecessary and avoidable harms to the necessary costs of dealing with a public health emergency.^1^

The lack of transparency by Australian authorities is exemplified by government failures to acknowledge independently identified limitations of pandemic responses.^2^ The imposition of a ban on in-person attendance by unvaccinated members at RANZCP events by the Board, announced August 11th and revoked on October 5th, provides an opportunity to gauge the Board’s commitment to good governance and transparency. The minimum standard for a decision that infringed one group of members’ rights to benefit others was a serious process to establish a strong probability that banning unvaccinated members would reduce infection of vaccinated members.

In personal communications the Board justified the policy by reference to ATAGI policy documents (RANZCP Board & Staff, pers. comms., 2022), but ATAGI reported well beforehand there was “insufficient evidence...to support vaccinating healthy workers in...healthcare settings solely on the grounds of reducing transmission to others.”^3^ The best contemporary evidence showed that after a small decrease in transmission following vaccination, protection rapidly waned “reaching levels that were similar to those in unvaccinated persons by 12 weeks.”^4^ As vaccination was mandatory for all Australian health workers before the start of 2022, the risk of Covid transmission by the majority of vaccinated RANZCP Fellows was the same as unvaccinated Fellows when the ban was introduced. There was no rational basis for the Board to conclude that there would be a benefit to vaccinated members from the exclusion of unvaccinated members from in-person meetings.

In personal communication with the President, President-elect, former and Acting CEOs, and three individual Board members, the only justification for the ban has been the reference to the ATAGI website which explicitly ruled out the reduction of transmission as a rationale for requiring Covid vaccination. The President has now declared that neither the Board nor RANZCP staff will engage in any further discussion of the matter, and the acting CEO has refused to discuss any alternative means of resolving the dispute (RANZCP Board & Staff, pers. comms., 2022). Given that the issue would at any time have been, and still could be, resolved by any evidence or policy justifying the in-person ban, this definitive refusal to engage does not suggest commitment to transparent governance or accountability to members.
